# Magnetization Reversal and Magnetic Anisotropy in Ordered CoNiP Nanowire Arrays: Effects of Wire Diameter

**DOI:** 10.3390/s150305687

**Published:** 2015-03-09

**Authors:** Luu Van Thiem, Le Tuan Tu, Manh-Huong Phan

**Affiliations:** 1 Faculty of Engineering Physics and Nanotechnology, VNU University of Engineering Technology, Vietnam National University, 144 Xuan Thuy, Cau Giay, Hanoi, Vietnam; E-Mail: hilvt22380@yahoo.com; 2 Faculty of Basic Science, Hanoi Industrial College for Textile, Garment and Fashion Le Chi, Gia Lam, Hanoi, Vietnam; 3 Faculty of Physics, VNU University of Science, Vietnam National University, 334 Nguyen Trai, Thanh Xuan, Hanoi, Vietnam; 4 Department of Physics, University of South Florida, Tampa, FL 33620, USA

**Keywords:** CoNiP nanowires, magnetic properties, electrodeposition, magnetic sensors

## Abstract

Ordered CoNiP nanowires with the same length of 4 µm and varying diameters (*d* = 100 nm–600 nm) were fabricated by electrodeposition of CoNiP onto polycarbonate templates. X-ray diffraction, scanning electron microscopy, and high-resolution transmission electron microscopy confirmed the quality of the fabricated nanowires. Magnetic measurements and theoretical analysis revealed that the magnetization reversal and magnetic anisotropy were significantly influenced by varying of the diameters of the nanowires. There existed a critical wire diameter (*d_c_* ≈ 276 nm), below which the magnetization reversal occurred via a coherent rotation mode, and above which the magnetization reversal occurred via a curling rotation mode. The easy axis of the magnetization tended to change in direction from parallel to perpendicular with respect to the wire axis as the wire diameter exceeded *d_c_* ≈ 276 nm. With increasing wire diameter, the coercive field (*H_c_*) and the remanent to saturation magnetization ratio (*M_r_*/*M_s_*) were also found to rapidly decrease in the range *d* = 100–400 nm and gradually decrease for *d* > 400 nm.

## 1. Introduction

High aspect-ratio magnetic nanomaterials, especially magnetic nanowire arrays have generated growing interest in the scientific community due to their potential applications in magnetic sensors, high-density magnetic recording, bioengineering, and magneto-electronic devices [1,2,3,4,5,6,7,8,9,10]. The magnetic nanowires possess unique properties that are quite different from those of their thin film, nanoparticle, and nanotube counterparts [6,9]. The magnetic nanowires possess quasi-one dimensional (1D) anisotropic structures along the wire axis, resulting in their anisotropic magnetic properties. The magnetic properties of the nanowires are governed by several material parameters, such as diameter, length, and composition [1,6,9]. It has been reported that the coercivity (*H_c_*), remanent magnetization (*M_r_*), and saturation magnetization (*M_s_*) are dependent on the direction of an externally applied field. There have been numerous reports on electrodeposited Co, Ni, and Fe nanowires and their alloys with large crystalline anisotropies [6,11,12,13,14,15]. Among them, CoNiP nanowires are of particular interest due to its larger *H_c_* and higher *M_s_* compared to their single metals [16]. Rani *et al.* reported that the CoNiP nanowires possessed *H_c_* as large as 500 Oe, and the crystal structure of the wire was a mixture of fcc and hcp [17]. The use of a nanoporous membrane is believed to increase the coercivity and squareness of the magnetic loops of the nanowires as compared to the thin film or the bulk material of the same composition [6,17]. If the length of a wire is much larger than its diameter, the easy axis of the magnetization tends to be aligned along the wire length due to its large shape anisotropy [1,6]. It has been shown that the magnetic properties of an arranged magnetic nanowire system are determined by the magnetostatic interaction among nanowires and the magnetic characteristic of individual nanowires, which is governed by its magnetic anisotropy (e.g., shape and magnetocrystalline anisotropies) [18,19,20]. Both the magnetostatic interaction and magnetic anisotropy have significant influences on the magnetization reversal process [19]. At room temperature, the magnetocrystalline anisotropy constant of bulk hcp cobalt was determined to be *K*_1_ = 5.0 × 10^6^ erg/cm^3^, while the shape anisotropy of magnetic nanowires was reported to be *K_s_* = π*M*^2^*_s_* = 6.10^6^ erg/cm^3^ [21]. From both the basic and applied research perspectives, a clear understanding of the effects of synthesis parameters (e.g., deposition current and time, pH solution) [22,23,24,25,26,27] and morphology (e.g., wire length and diameter) [6,9,28,29] on the magnetic properties of ordered nanowire arrays is essential. Despite some previous efforts [16,17], effects of the wire diameter on the magnetic properties of the ordered CoNiP nanowire arrays remained to be investigated.

In this paper, we investigated the effects of varying diameter (*d* = 100–600 nm) on the magnetic properties of ordered CoNiP nanowires with the same length of 4 µm, which were electrodeposited into polycarbonate templates. We found that the magnetization reversal was significantly influenced by varying the diameter of the nanowire, and that there existed a critical wire diameter (*d_c_* ≈ 278 nm) below and above which the magnetization mechanisms were different. The easy axis of the magnetization changed in direction from parallel to perpendicular with respect to the wire axis, when the wire diameter exceeded *d_c_* ≈ 278 nm. These findings are of practical importance in exploiting ordered CoNiP nanowire arrays for use in advanced sensor and magneto-electronic devices.

## 2. Experimental Section

In this work, porous polycarbonate templates with the pore diameters of 100, 200, 400, and 600 nm and a thickness of 3 µm were used. The interpore distances in the templates were ~50 nm. The polycarbonate templates were purchased from Whatman (Whatman^®^ Nuclepore Track-Etched Membranes, Seoul, Korea). Before electrodeposition, a copper (Cu) layer of the thickness of about 100 nm was sputtered onto one side of the polycarbonate template and used as the working electrode to fabricate magnetic nanowires. Afterward, the polycarbonate template was placed in an electrolytic bath. A three-electrode bath was used for electrochemical experiments. An Ag/AgCl electrode was used as the reference electrode (*RE*), the counter electrode was a platinum mesh (*CE*), and the working electrode (*WE*). The electrolyte used to electro-deposit the CoNiP nanowires had the following compositions: 0.2 M CoCl_2_·6H_2_O, 0.2 M NiCl_2_·6H_2_O, 0.25 M NaH_2_PO_2_, 0.7 M H_3_BO_3_, and 0.001 M saccharin. The deposition potential was −0.9 V, while the pH value of the electrolyte bath was 5.1. The deposition potential of −0.9 V was chosen based on the obtained result of cyclic voltammetry (CV) for the trinary CoNiP system. In this voltammogram, we observed that deposition started at −0.5 V until −1.2 V for a negative sweep and there was a reduction peak at −0.9 V. The electrodeposition process was performed at room temperature. The morphology of the CoNiP nanowires was investigated by scanning electron microscopy (SEM, JSM-5410LV, JEOL Ltd., Tokyo, Japan), transmission electron microscopy (TEM, Tecnai G^2^ 20 S-TWIN, FEI, Oregon, USA) equipped with high-resolution TEM (HR-TEM) and selected area electron diffraction (SAED). The nominal composition of the nanowires was determined by energy dispersive spectroscopy (EDS). The crystal structure was analyzed by X-ray diffraction (XRD, Advance D8, Bruker, Karlsruhe, Germany). Magnetic hysteresis loops (M-H) were recorded at room temperature using a vibrating sample magnetometer (VSM 7404, Lake Shore, OH, USA) in fields up to 10 kOe. The magnetic field was applied parallel and perpendicular to the wire axis.

## 3. Results and Discussion

**Figure 1:**
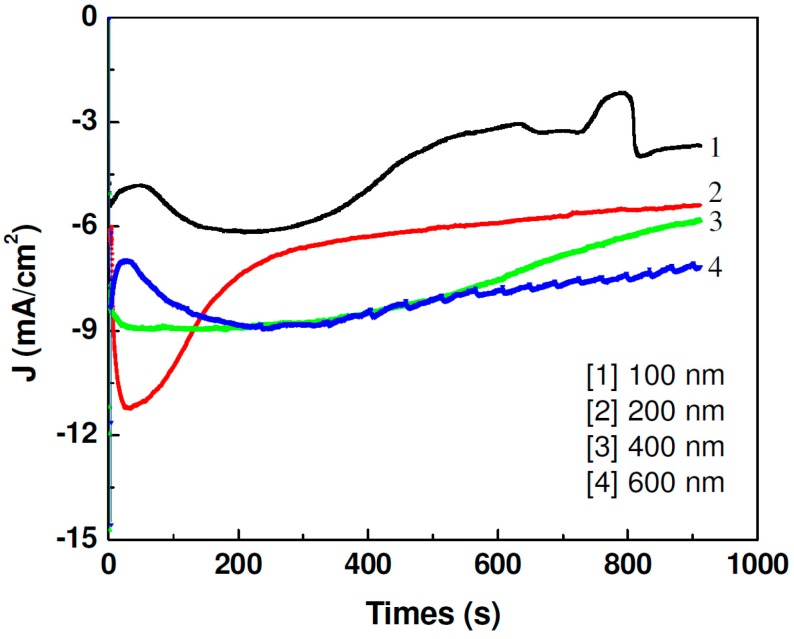
Current density *versus* time plots of CoNiP electrodeposited solution at a constant potential of −0.9 V. In this plot, 100 nm, 200 nm, 400 nm, and 600 nm correspond to the diameters of the nanowires.

The deposition process was done while monitoring the current density—time profiles to derive information related to the nanowires’ growth mechanism. Figure 1 shows the current density behavior as a function of wire diameter during wire deposition.

Electrodeposition curves were obtained in an electrolyte at a constant potential of −0.9 V with respect to the Ag/AgCl reference electrode. The current density *versus* time plots clearly showed that the nanowire deposition was not a steady state process. When the pores were empty, the deposition current dropped suddenly and reached an almost steady state when the wires were growing. The current density gradually increased with the filling of the pores and reached a steady state when the wires reached the top of the membrane. As the deposition continued, the deposition area was constant and the resulting current density was constant.

**Figure 2:**
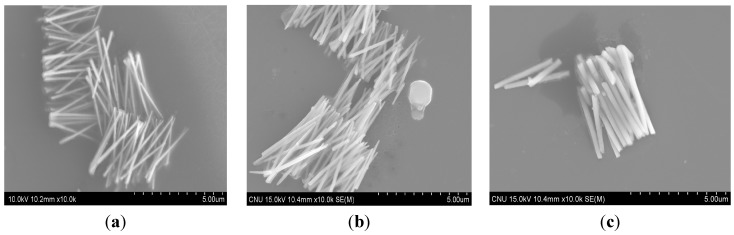
SEM images of CoNiP nanowires with different diameters: (**a**) 100 nm; (**b**) 200 nm; and (**c**) 400 nm.

Figure 2 shows some typical SEM images of CoNiP nanowires released from the polycarbonate templates. The diameter (*d*) of the nanowires was determined to be about 100, 200, 400 and 600 nm, respectively. The length of the nanowires was determined to be about 4 µm. It is worth noting here that the fabricated nanowires are compact and uniform with high-aspect ratios.

**Figure 3:**
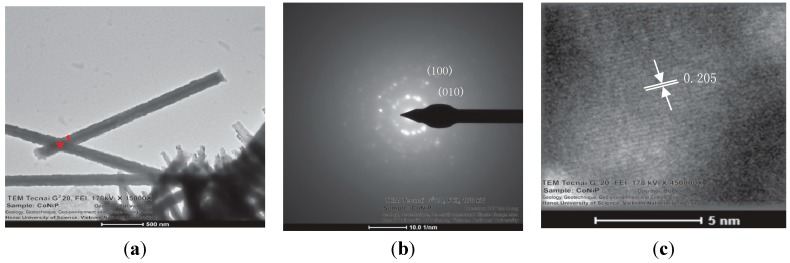
(**a**) A typical TEM image and (**b**) a SAED image of the 200 nm CoNiP nanowires; (**c**) an HRTEM micrograph of a single CoNiP nanowire.

The microstructures of the CoNiP nanowires were further analyzed by TEM and HR-TEM. Figure 3a shows a typical TEM image of the 200 nm CoNiP nanowires. It can be observed that the nanowires are continuous with a uniform diameter. The SAED pattern indicated the polycrystalline structure of the nanowires (Figure 3b). This was further confirmed by an HR-TEM image as shown in Figure 3c. The lattice space was determined to be about 0.205 nm, corresponding to the (002) plane of the hcp CoNiP phase [30].

**Figure 4:**
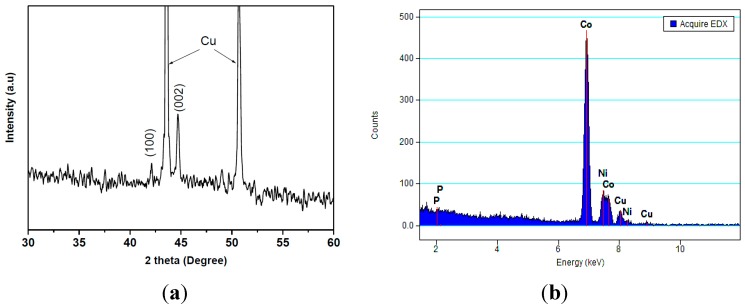
(**a**) XRD pattern and (**b**) EDS spectrum of the 200 nm CoNiP nanowires.

Figure 4a shows the XRD pattern of the 200 nm CoNiP nanowires after removing from the polycarbonate template. It can be seen that the positions of diffraction peaks are 42.07° and 44.93°, corresponding to (100) and (002) with the pH value of 5.1. This indicates that the diffraction peaks of CoNiP phases correspond to the hexagonal close packed (hcp) with a preferred crystallographic c-axis orientation parallel to the wire axis. As can be seen in Figure 4a, the (002) peak is intense compared to the (100) peak. This result is consistent with that was previously reported by Park *et al.* [26]. The copper (Cu) peaks appeared to occur due to the copper film sputtered on the surface of the polycarbonate template. We have checked the XRD for all the nanowires and found no significant change in the structure with varying the wire diameter from 100 nm to 600 nm.

Figure 4b shows an EDS spectrum of the 200 nm CoNiP nanowires. It is observed that the CoNiP nanowires contained only Co, Ni and P elements. The presence of copper (Cu) peaks is due to the copper film sputtered on the surface of the sample. According to the EDS analysis, the atomic percentages of Co, Ni, and P were determined to be 81.07%, 12.68%, and 6.25%, respectively. We have checked the composition of CoNiP alloy in the synthesized nanowires using EDS and found that the composition did not change significantly with varying the wire diameter.

**Figure 5:**
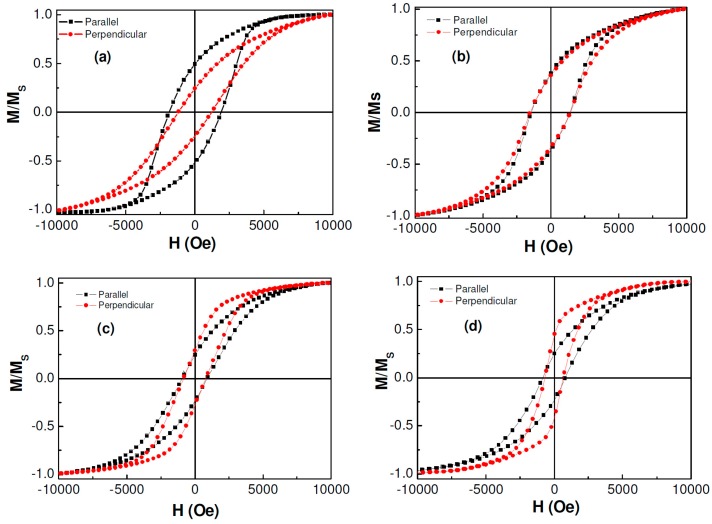
Magnetic hysteresis loops of CoNiP nanowires with different diameters of (**a**) 100 nm; (**b**) 200 nm; (**c**) 400 nm; and (**d**) 600 nm measured at room temperature.

**Figure 6:**
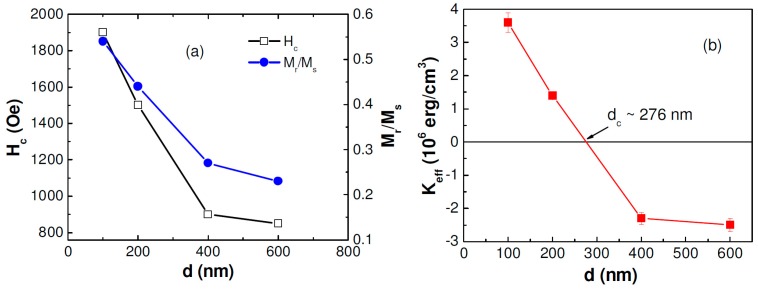
(**a**) Wire-diameter dependence of the coercive field (*H_c_*) and the remanence to saturation magnetization ratio (*M_r_*/*M_s_*) for the magnetic field applied parallel to the nanowire axis; (**b**) wire-diameter dependence of the effective anisotropy constant (*K_eff_*) of ordered CoNiP nanowires.

Once the nanowires were well structurally characterized, we performed magnetic measurements on them. For all magnetic measurements, the nanowires were kept in the membrane, and magnetic field was applied parallel and perpendicular to the wire axis. Figure 5a–d shows the room temperature hysteresis loops (M-H) of ordered CoNiP nanowire arrays with different diameters. For both parallel and perpendicular configurations, the coercive field (*H_c_*) and the remanent to saturation magnetization ratio (*M_r_*/*M_s_*) decreased with an increase in the wire diameter (*d*), as seen in Figure 6a. A decreasing trend in *H_c_* with increasing *d* was also reported for Co, Ni, and Fe nanowires [6].

In order to understand the effects of varying wire diameter on the magnetization reversal of the present CoNiP nanowires, we have employed a two-magnetization reversal processes model [31]. According to this model, there exists a critical radius *R_c_* for a transition between the two-magnetization reversal processes. If *R* < *R_c_*, the magnetization reversal occurs via a coherent rotation mode, and if *R* > *R_c_* the magnetization reversal occurs by a curling rotation mode [7,19]. *R_c_* can be determined when the external magnetic field is applied along the easy magnetization axis [19].

(1) $$R_{C}=q{\left(\frac{2}{N_{\;a}}\right)}^{\frac{1}{2}}\times \frac{A^{\frac{1}{2}}}{M_{\;s}}$$

where *q* is a constant depending on the length/diameter ratio of the particles and it is in the range of 1.8412 for an infinite cylinder (used in this study) to 2.0816 for a sphere. *N_a_* is the demagnetization factor (2π for a cylinder). *A* is the constant of exchange stiffness (erg/cm) and it is in the range of 1.0 × 10^−6^ to 1.3 × 10^−6^ (erg/cm). *M_s_* is the saturation magnetization (emu/cm^3^). Using the *M_s_* of the CoNiP nanowires, *R_c_* was determined to be ~138 nm. This suggested that for CoNiP nanowires with *R* < 138 nm (the equivalent wire diameter, *d* = 276 nm), the magnetization reversal occurred via a localized coherent process. Meanwhile, for CoNiP nanowires with *R* > 138 nm (*d* > 276 nm) the curling rotation mode governed the magnetization reversal process. While the used model reasonably interprets the wire diameter-dependent magnetization reversal in our CoNiP nanowires, we note that other magnetization processes like transverse domain wall apart from curling may also be significant, especially for CoNiP nanowires with *d* > 276 nm. An excellent experimental and theoretical analysis on the magnetization reversal mechanisms that govern the magnetic properties of ferromagnetic nanowires depending on their aspect ratio has been made by Ross *et al.* [32].

Another feature to be noted in Figure 5 is that with increasing wire diameter the easy axis of the magnetization tended to change in direction from parallel to perpendicular with respect to the wire axis. This indicated a remarkable change in magnetic anisotropy in these nanowires, as the wire diameter was altered. To quantitatively understand this, we have calculated the effective anisotropy constant *K_eff_* of the nanowires using the method [20]:

(2) $$K_{\;eff}=2\;\pi \;M_{\;s}\left(H_{1}^{s}-H_{2}^{s}\right)$$

where *M_s_* is the saturation magnetization, $H_{1}^{s}$ is the value of the applied magnetic field needed to saturate the magnetization perpendicular to the nanowires axis, and $H_{2}^{s}$ is the value of the applied magnetic field needed to saturate the magnetization parallel to the wire axis. Figure 6b shows an evolution of *K_eff_* as a function of wire diameter. It can be seen that *K_eff_* decreased with increasing the wire diameter. In the case of *K_eff_* > 0, the nanowires with smaller diameters (*d* < 276 nm), the easy axis of the magnetization was parallel to the wire axis, while in the case of *K_eff_* < 0, the nanowires with larger diameters (*d* > 276 nm), the easy axis of the magnetization was perpendicular to the wire axis [8]. This trend is similar to that reported previously by Vazquez *et al.* [29], where the easy axis of the magnetization of the electrodeposited Ni nanowires was shown to rotate from the axial to transverse direction in the plane of the membrane, when the diameter of the nanowire exceeded a critical value. In this case, the nanowires with diameters smaller than the critical value prefer to have the easy axis of the magnetization along the wire axis, due to the dominance of shape anisotropy. Meanwhile, the magnetic moments of the larger nanowires tend to align in the perpendicular direction with respect to the wire axis, resulting from enhanced magnetostatic interactions between the nanowires [8,29,32].

Finally, we note that the tunable magnetic properties of CoNiP nanowires make them very promising for a wide variety of applications, such as nanowire read sensors based on the magneto-resistance effect [33]. Nanodevices using such magnetic nanowires can also be a useful platform for sensitive detection of biological and chemical species. It has recently shown that magnetic nanowires are a very attractive candidate material for use in magnetic hyperthermia for cancer treatment, in magnetic separation, and in targeted drug delivery [34].

## 4. Conclusions

The ordered CoNiP nanowire arrays with varying wire diameters (100 nm–600 nm) were successfully fabricated by the electrodeposition method. Magnetic measurements and analysis revealed the strong influences of wire diameter on the magnetization reversal processes and magnetic anisotropy of the nanowires. For CoNiP nanowires with diameters smaller than 276 nm, the magnetization reversal occurred via the coherent rotation mode, while for CoNiP nanowires with diameters greater than 276 nm the magnetization reversal occurred via the curling rotation mode. The easy axis of the magnetization changed in direction from parallel to perpendicular with respect to the wire axis, when the wire diameter exceeded a critical value of ~276 nm.
